# Multiple presentations of drug-resistant central nervous system tuberculosis: a case report

**DOI:** 10.1097/MS9.0000000000005591

**Published:** 2026-08-26

**Authors:** Saeed Soleiman-Meigooni

**Affiliations:** Department of Infectious Diseases, Faculty of Medicine, Infectious Diseases Research Center, Aja University of Medical Sciences, Tehran, Iran

**Keywords:** case report, central nervous system tuberculosis, Guillain–Barré syndrome, tuberculoma, tuberculous immune reconstitution inflammatory syndrome, tuberculous ventriculitis

## Abstract

**Introduction and importance:**

Central nervous system tuberculosis (CNS-TB) is a severe manifestation of *Mycobacterium tuberculosis* infection that can cause substantial morbidity and mortality. Tuberculous ventriculitis and intramedullary tuberculoma are two rare complications, and their coexistence poses significant diagnostic and therapeutic challenges.

**Case presentation:**

A 19-year-old woman with a history of systemic lupus erythematosus, treated with prednisolone and methotrexate for the past 2 years, was diagnosed with isoniazid-resistant tuberculous meningitis and multiple intracranial tuberculomas. After 4 weeks of antitubercular treatment (ATT), she experienced progressive quadriparesis, lethargy, seizures, and fever. Brain and cervical spine MRI revealed tuberculous ventriculitis and a C3-C5 intramedullary tuberculoma. The ATT led to a paradoxical neurological decline and the emergence of new lesions, consistent with tuberculous immune reconstitution inflammatory syndrome (TB-IRIS). Additionally, electrodiagnostic studies indicated the possibility of Guillain–Barré syndrome (GBS). She was referred for cervical spinal cord decompression while continuing isoniazid-sparing ATT and high-dose corticosteroids. Her neurological condition improved, and a follow-up MRI conducted 2 years later showed radiologic resolution.

**Clinical discussion:**

This case underscores the diagnostic challenges of CNS-TB in an immunocompromised patient, particularly when TB-IRIS, ventriculitis, intramedullary tuberculoma, and GBS co-occur. A positive outcome required timely identification of paradoxical worsening and immunological reactions, meticulous exclusion of alternative diagnoses, and integrated medical and surgical interventions.

**Conclusion:**

The emergence of new neurological lesions after the initiation of ATT in patients with CNS-TB, especially those receiving immunosuppressive therapy, necessitates consideration of TB-IRIS. Prompt imaging, evaluation of alternative diagnoses, and interdisciplinary management may improve outcomes.

## Introduction

Central nervous system tuberculosis (CNS-TB) constitutes approximately 1% of all tuberculosis cases; however, it results in mortality and morbidity in nearly half of patients^[^1,2^]^. The rarity, insidious onset, and often slow progression of this entity frequently overlap with other central nervous system (CNS) diseases and usually lead to delayed diagnosis and higher rates of adverse events and worse outcomes. Furthermore, drug resistance, as reported in several cases, is another concern in CNS-TB^[^3,4^]^. This drug resistance complicates treatment regimens and can result in treatment failure, worsening patient outcomes, and increased healthcare costs.

HIGHLIGHTS

- A rare case of drug-resistant tuberculous meningitis and intracranial tuberculomas.
- Occurrence of spinal tuberculoma, Guillain–Barré syndrome, and tuberculous ventriculitis following tuberculous immune reconstitution inflammatory syndrome.
- The patient developed drug-induced hepatitis and a healthcare-associated infection during treatment.
- Successful management resulted in complete resolution of tuberculomas and ventriculitis.
- The need for a meta-analysis of tuberculous ventriculitis and drug-induced hepatitis in central nervous system tuberculosis.

Hydrocephalus remains the most common complication associated with CNS-TB. However, tuberculous immune reconstitution inflammatory syndrome (TB-IRIS) is a relatively frequent complication that can cause existing tubercles to expand or rupture, or lead to the emergence of new lesions in other areas of the CNS during treatment^[^5,6^]^. Intramedullary tuberculoma is an uncommon, debilitating presentation of CNS-TB that may develop after TB-IRIS and induce neurological deficits below the level of the lesion; however, it can be fatal if it expands within the cervical medulla. Surgical cord decompression is typically required for treatment, in conjunction with antitubercular treatment (ATT)^[^7,8^]^. Nevertheless, there is no consensus regarding the management of another complication, tuberculous ventriculitis.

Tuberculous ventriculitis is an infrequent and devastating complication of CNS-TB that typically occurs following TB-IRIS and the expansion of a tubercle into the ventricular system^[^6,9^]^. This paper discusses an immunocompromised female patient with drug-resistant CNS-TB who initially exhibited tuberculous meningitis (TBM) and multiple intracranial tuberculomas, later progressing to TB-IRIS, an intramedullary tuberculoma, and ventriculitis. The author outlines the clinical presentation, treatment course, complications, and prognosis of the patient in accordance with the SCARE-2025 checklist^[^10^]^.

## Case presentation

### History and physical examination

A 19-year-old female with a 2-year history of systemic lupus erythematosus (SLE) was referred to our emergency department at a tertiary hospital, presenting with a 4-day history of headache, high-grade fever, and several hours of lethargy and progressive quadriparesis. She was diagnosed with TBM and multiple intracranial tuberculomas at a different hospital 4 weeks earlier and had undergone ATT. Her SLE had remained clinically stable on maintenance immunosuppressive therapy with prednisolone and methotrexate, with no recent exacerbations. She had no recent history of psychotic behavior, illicit drug use, alcohol consumption, or smoking. The cerebrospinal fluid (CSF) analysis at the same hospital indicated a protein concentration of 83 mg/dL, a white blood cell count of 211 cells/mm^3^, and a glucose level of 44 mg/dL. In addition, the diagnosis of TBM was confirmed by a positive CSF polymerase chain reaction (PCR) for *Mycobacterium tuberculosis*. Moreover, testing demonstrated resistance to isoniazid (INH) through KatG gene analysis.

Six hours before her arrival at our hospital, her condition had abruptly deteriorated, marked by the onset of generalized tonic-clonic seizures and tachypnea. Upon admission, she presented with a toxic appearance, with a temperature of 39.3°C, a pulse rate of 120 beats per minute, a respiratory rate of 24 breaths per minute, and a blood pressure of 103/58 mmHg. A mildly comatose state (Glasgow Coma Scale score of 11) accompanied by photophobia, neck stiffness, and flaccid quadriparesis was observed during the physical examination. Both the upper and lower extremities demonstrated reduced deep tendon reflexes; however, she displayed normal pupillary reflexes, intact extraocular movements, and symmetric facial muscle tone.

### Laboratory findings

Initial laboratory investigations indicated a hemoglobin level of 9.5 g/dL, a platelet count of 148 000/mm^3^, and a white blood cell count of 4800/mm^3^. Serum electrolyte levels, liver function tests, and blood cultures were within normal limits. The erythrocyte sedimentation rate (ESR) was 91 mm/h, while the C-reactive protein (CRP) level was 165 mg/dL. Additional diagnostic tests, including anti-nuclear antibody, anti-double-stranded DNA, C3, C4, and CH50, were performed to evaluate for a SLE flare-up, and all results were within normal limits. Furthermore, CNS infections, such as HIV infection, cysticercosis, toxoplasmosis, and echinococcosis, were ruled out using an enzyme-linked immunosorbent assay (ELISA).

### Imaging

**Figure 1.**
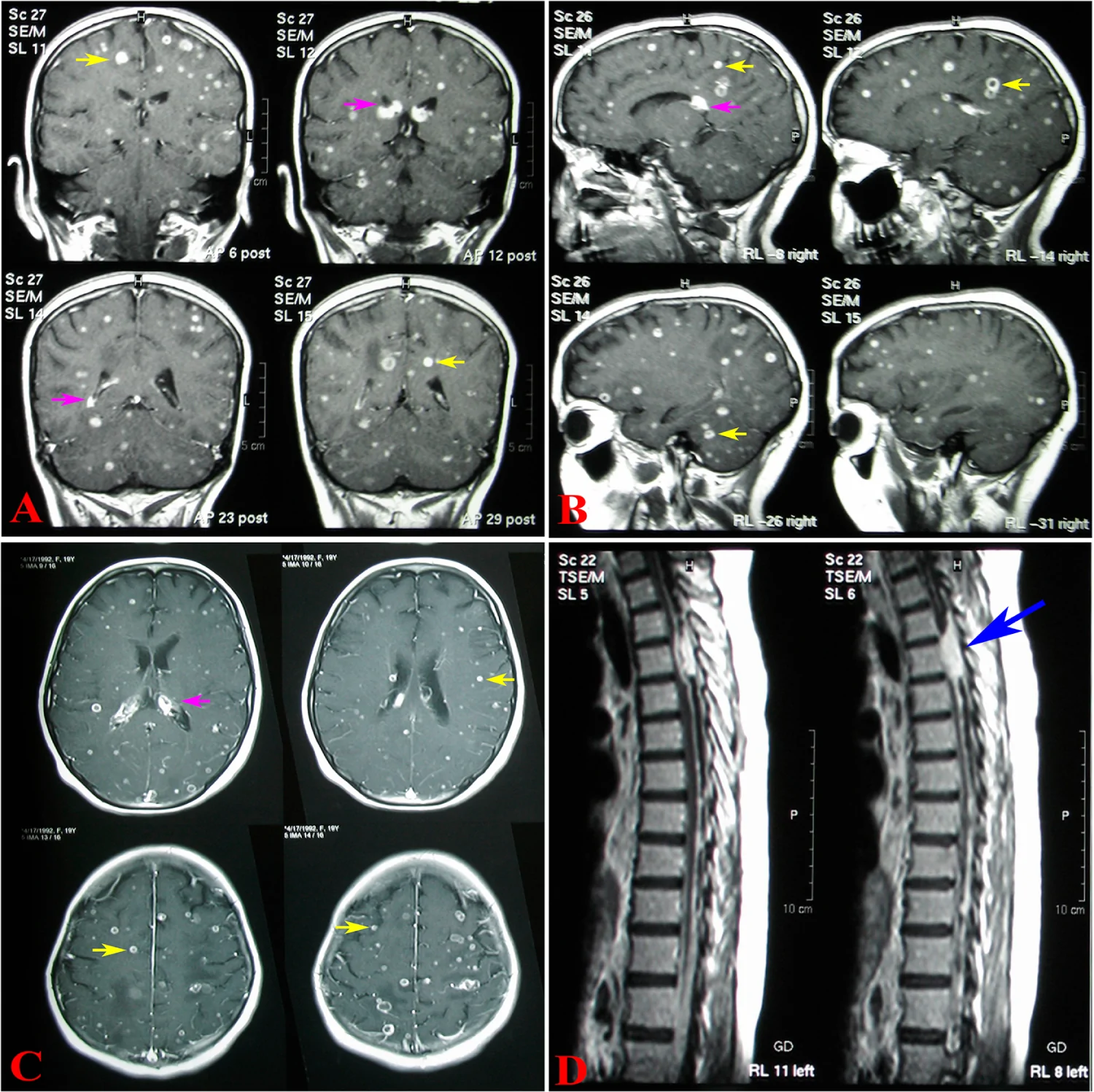
Multiple round hyper signal foci with peripheral enhancement and surrounding edema in the cerebral hemispheres and cerebellum (yellow arrows), compatible with tuberculoma; ependymal enhancement and ventricular sludge (magenta arrows), compatible with ventriculitis, in T1-weighted MRI: coronal (A), sagittal (B), and horizontal (C) views. Dural enhancement and hypersignal foci from C3 to C5 (blue arrow), compatible with intramedullary tuberculoma (D).

The T1-weighted brain magnetic resonance imaging (MRI) revealed multiple hyperintense lesions with peripheral enhancement and surrounding edema in both cerebral hemispheres and the cerebellum, consistent with intracranial tuberculomas. Additionally, there was intraventricular sludge and ependymal enhancement, characteristic of tuberculous ventriculitis (Fig. 1A–C). The cervical spinal MRI showed dural enhancement and hyperintense foci from C3 to C5, consistent with an intramedullary tuberculoma (Fig. 1D). These novel findings of intramedullary tuberculoma and ventriculitis were not documented at the time of the initial TBM diagnosis. Therefore, the patient was diagnosed with tuberculosis-associated immune reconstitution inflammatory syndrome (TB-IRIS) following the initiation of appropriate ATT. This diagnosis was based on the temporal relationship between clinical deterioration and the emergence of new CNS lesions, as well as the exclusion of alternative diagnoses, including CNS lupus, other infections, or malignancy.

### Neuromuscular diagnostic study

Electromyography and nerve conduction velocity (EMG/NCV) studies, conducted to evaluate quadriparesis, revealed severe mixed axonal and demyelinating motor polyneuropathy in the lower limbs, along with moderate mixed axonal and demyelinating motor polyneuropathy in the upper limbs. These findings were consistent with Guillain–Barré syndrome (GBS).

### Management

The initial management consisted of hydration, anticonvulsants, and the continuation of an INH-sparing ATT regimen that included rifampin, ethambutol, pyrazinamide, levofloxacin, and amikacin. The physician discontinued prednisolone and methotrexate, initiating high-dose dexamethasone at 8 mg three times daily. Subsequently, the patient was transferred for cervical spinal cord decompression because of an intramedullary tuberculoma and quadriparesis, following discussions with her first-degree relatives.

The procedure was conducted under general anesthesia, with the patient positioned prone. A midline posterior cervical incision was made over the C3–C5 vertebrae, followed by subperiosteal dissection to expose the posterior cervical vertebrae. The dura mater was meticulously opened under a microscope, and the arachnoid was dissected to reveal the spinal cord. A midline myelotomy at C3–C5 was performed to debulk the granulomatous lesion while preserving normal neural tissue. A whitish-gray intradural mass was excised and sent for histopathological examination, which indicated necrotizing inflammation, caseous granuloma, and mononuclear infiltration.

### Complications

She developed drug-induced hepatitis during the second week of her admission (the sixth week of ATT), with symptoms of vomiting and transaminase elevations of more than threefold, necessitating the temporary withholding of hepatotoxic drugs such as rifampin and pyrazinamide until her transaminase levels returned to normal. Additionally, during the third week of her admission, she developed a healthcare-associated infection, which presented with dyspnea, a purulent cough, and a positive procalcitonin test. This infection was managed by adding linezolid and meropenem to her treatment regimen.

### Outcome and follow-up

**Figure 2.**
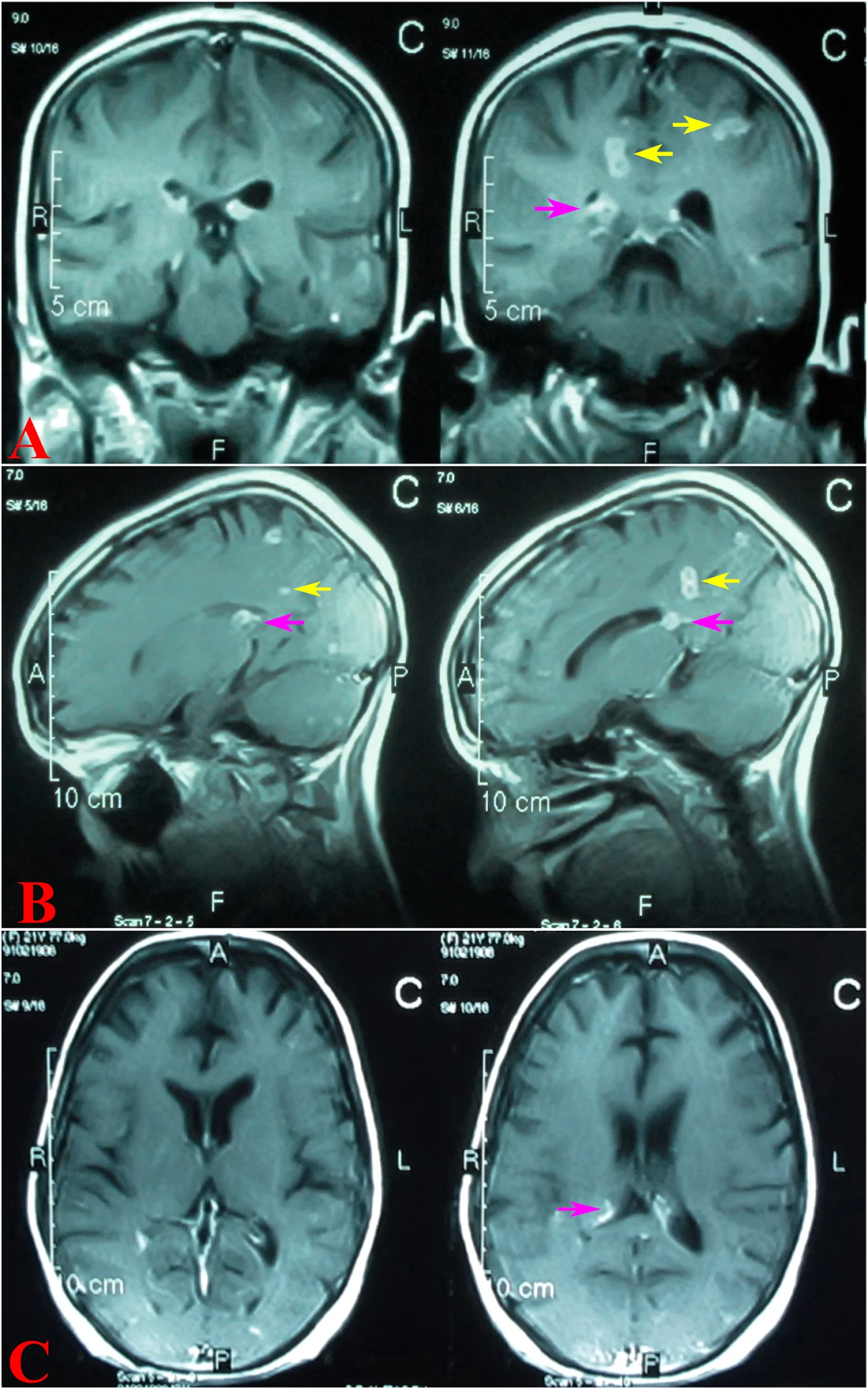
Brain MRI 1 year after treatment revealed the disappearance of many intracranial tuberculomas (yellow arrows), as well as partial recovery of ventriculitis (magenta arrows) in T1-weighted MRI: coronal (A), sagittal (B), and horizontal (C) views.

Throughout all stages of treatment, the patient and her family cooperated well with the medical team. Her neurological status gradually improved over the following weeks, and she was discharged on day 35. A four-drug ATT regimen, which included rifampin, ethambutol, pyrazinamide, and levofloxacin, was continued for 1 year. The physician also prescribed oral prednisolone at a dosage of 25 mg daily for 2 months, then tapered to 10 mg daily for the remainder of the treatment. A follow-up MRI at 1 year, as shown in Fig. 2, revealed complete resolution of the intramedullary tuberculoma and most intracranial tuberculomas, along with partial improvement in ventriculitis. As a result, ATT was extended for another year. After 2 years, the patient was still stable, with muscle strength of 4/5 in the upper limbs and 3/5 in the lower limbs. Follow-up MRI indicated complete radiologic resolution. No relapse was observed during six in-person visits throughout the 2-year follow-up period. Table 1 provides a full chronological timeline of clinical events and interventions, and Table 2 summarizes key laboratory findings.

**Table 1:** Chronological timeline of clinical events and interventions.

Time point	Clinical events and findings	Key investigations	Management
2 years before the current admission	Diagnosis of SLE; stable on prednisolone and methotrexate	–	Maintenance immunosuppressive therapy
4 weeks before the current admission	Diagnosis of TBM and multiple intracranial tuberculomas at another hospital	CSF: protein 83 mg/dL, WBC 211/mm^3^, glucose 44 mg/dL; positive CSF PCR for M. tuberculosis; INH resistance (KatG)	ATT initiated (INH-sparing regimen)
Day 4	Onset of fever, headache, lethargy, progressive limb weakness	–	Outpatient symptomatic management
Day 0 (Emergency Department)	High fever, seizures, tachypnea, decreased consciousness, quadriparesis, neck stiffness	CBC, Biochemistry; Microbiology, Immunology, and Inflammatory biomarkers, Brain and Cervical MRI	Airway protection, anticonvulsants, continuation of ATT
Day 1	Identification of new intramedullary tuberculoma and ventriculitis; diagnosis of TB-IRIS and GBS	EMG/NCV	High-dose dexamethasone started; neurosurgical consultation
Early hospital course	Progressive neurological deficit related to an intramedullary lesion	–	Spinal surgery and cervical spinal cord decompression and lesion excision
Week 2 after admission (6th week after ATT)	Drug-induced hepatitis	Liver function tests	Temporary cessation of rifampin and pyrazinamide
Week 3 after admission	Healthcare-associated infection	Microbiology tests, HAI work-up	Linezolid and meropenem added
Hospital day 35	Significant clinical improvement; able to be discharged	–	Discharge on rifampin, ethambutol, pyrazinamide, and levofloxacin for 1 year
12 months after ATT initiation	Sustained clinical recovery	Brain and spinal MRI	Complete resolution of intramedullary lesion, but partial improvement of ventriculitis; ATT extended to an additional year
24 months after ATT initiation	Stable neurological function (UL 4/5, LL 3/5) with no relapse	Brain and spinal MRI	Complete radiologic resolution; end of ATT and follow-up

**Table 2:** Summary of key laboratory findings.

Time point	Test/parameter	Result/description
At initial TBM diagnosis (4 weeks before admission)	CSF Analysis	CSF WBC = 211 cells/mm^3^
		CSF Protein =83 mg/dL
		CSF Glucose =44 mg/dL
		Positive CSF PCR for *M. tuberculosis*
	Drug resistance testing	INH resistance (*KatG* gene mutation)
On admission	CBC	Hb = 9.5 mg/dL, WBC = 4800 cells/mm^3^, Plt = 148 000/mm^3^
	Inflammatory biomarkers	ESR =91 and CRP = 165 mg/dl, normal rheumatology tests
	LFT and Electrolytes	Within normal limits
Week 2 after admission (6th week after ATT)	Liver function tests	Elevated ALT and AST, consistent with drug-induced hepatitis
Week 3 after admission	Microbiology tests, Procalcitonin	Positive Procalcitonin
During hospitalization	EMG/NCV	Severe mixed axonal and demyelinating motor polyneuropathy in lower limbs; moderate mixed axonal and demyelinating motor polyneuropathy in upper limbs

## Discussion

This case highlights a complex and rare progression of CNS tuberculosis in an immunocompromised young woman, characterized by TBM that was followed by paradoxical clinical deterioration, new intracranial and spinal lesions, and electrophysiological evidence of GBS. It is remarkable not only for the simultaneous presence of TBM, multiple intracranial tuberculomas, tuberculous ventriculitis, and an intramedullary tuberculoma, but also for the diagnostic difficulties posed by the underlying SLE and the continuation of immunosuppressive therapy. The chronological sequence of events, along with the radiologic, microbiologic, pathologic, and treatment-response data, strongly suggests that TB-IRIS complicates CNS tuberculosis.

Intramedullary tuberculoma represents a rare form of CNS-TB, which occurs in conjunction with disseminated tuberculosis or intracranial tuberculomas. This condition constitutes only 0.2–0.5% of all CNS tuberculomas, and its occurrence alongside intracranial tuberculoma is exceedingly rare. In most cases of intramedullary tuberculoma, ATT and surgical decompression of the spinal cord are used to stop neurological deficits from worsening^[^11–13^]^. Early surgical intervention, before irreversible spinal cord injury occurs, combined with ATT and corticosteroids, can lead to substantial neurological recovery^[^14,15^]^. In the case discussed, cervical MRI identified an intramedullary lesion spanning from C3 to C5; the patient exhibited rapidly progressive quadriparesis, characterized by both upper and lower motor deficits. As a result, surgical decompression was necessary for both neurological intervention and tissue diagnosis. The histopathological study revealed granulomatous inflammation with caseous necrosis, strongly confirming the presence of a tuberculoma and warranting the continuation of ATT.

Tuberculous ventriculitis is an uncommon and often under-recognized complication of CNS-TB. The incidence of this complication is estimated to be approximately 2.1%–2.9% among such patients^[^16,17^]^. In practice, ventriculitis may be overlooked because its clinical manifestations overlap with those of severe TBM. Common symptoms of ventriculitis include fever, decreased consciousness, neck stiffness, elevated intracranial pressure, seizures, and headache. CSF analysis often resembles that of TBM, showing elevated protein levels, hypoglycorrhachia, and a mild leukocyte count. MRI findings may include ependymal enhancement, ventricular sludge, hydrocephalus, and, occasionally, intraventricular pyogenic contents^[^1,6,9,17–19^]^. In this patient, the emergence of ependymal enhancement and ventricular sludge after 4 weeks of treatment, which were not present at the time of the initial diagnosis, strongly suggested a paradoxical inflammatory response. Although there is no standardized treatment guideline, prolonged ATT and high-dose corticosteroids are the primary treatments for patients with tuberculous ventriculitis.

Additionally, there are several reports of drug resistance among these patients that should be considered before starting ATT, as this resistance can complicate treatment outcomes and may necessitate alternative therapeutic strategies^[^16–18^]^. Furthermore, many patients continue to need neurosurgical intervention, particularly for symptomatic hydrocephalus or refractory elevated intracranial pressure.

TB-IRIS is likely the cause of the patient’s neurological deterioration after treatment began. This condition is becoming more recognized in cases of CNS-TB and can present as enlarging tuberculomas, worsening meningitis, or, less commonly, as ventriculitis and spinal involvement^[^1,20,21^]^. The diagnosis was based on the temporal relationship between ATT and clinical deterioration, the emergence of new CNS lesions despite ongoing therapy, and the absence of a more convincing competing diagnosis. The mortality rate associated with CNS TB-IRIS is estimated to range from 13% to 30%^[^21^]^.

Recent studies indicate that 12–20% of patients with CNS-TB may exhibit resistance to at least one anti-tuberculosis medication. The management of drug-resistant CNS-TB is further complicated by the limited penetration of second-line agents into the CNS, delays in identifying resistance, and the absence of standardized treatment protocols. Early drug-susceptibility testing, as demonstrated in our case, is vital for informing optimal treatment strategies and enhancing patient outcomes^[^22^]^.

An SLE flare with CNS involvement was an important consideration in the differential diagnosis for this patient. However, the absence of related clinical symptoms, such as psychotic symptoms, along with normal results from SLE-related tests, reduces the likelihood of this diagnosis. Additionally, confirmation of CNS TB is supported by a positive PCR test for *M. tuberculosis*, typical MRI findings indicative of ventriculitis, the identification of a caseating granuloma in the histopathological examination of the intramedullary cervical mass, and clinical improvement following ATT. Immunocompromised status heightens vulnerability to tuberculosis. Nonetheless, researchers have yet to comprehensively elucidate the specific types and severity of inflammatory responses in these patients following tuberculosis infection.

The electrophysiologic studies in the patient were consistent with GBS, which is an uncommon but recognized immune-mediated neurologic complication in the context of tuberculosis. In this case, the acute onset of flaccid quadriparesis, reduced deep tendon reflexes, and EMG/NCV evidence of mixed axonal and demyelinating motor polyneuropathy supported the diagnosis. The improvement observed without intravenous immunoglobulin suggests that control of the underlying infection and inflammatory burden may have contributed to neurologic recovery, although a direct causal relationship cannot be established from a single case.

The case also emphasizes the importance of anticipating treatment-related adverse events during prolonged ATT. Drug-induced hepatitis required the temporary interruption of rifampin and pyrazinamide, and a healthcare-associated infection, especially in critically ill patients, necessitated escalation of antimicrobial therapy. If healthcare professionals do not promptly recognize such complications, they can obscure the clinical picture and delay recovery. Close biochemical monitoring and multidisciplinary coordination are, therefore, essential in complicated CNS-TB, particularly when prolonged therapy, corticosteroids, and neurosurgical intervention are required.

Overall, this case contributes to the limited literature on the rare complications associated with CNS-TB, particularly tuberculous ventriculitis and intramedullary tuberculoma, which can arise in the context of TB-IRIS and autoimmune disease. It highlights several important lessons: any new neurological deterioration following the start of appropriate ATT should raise suspicion for TB-IRIS; CNS lupus should be considered in the differential diagnosis in patients with SLE; and timely MRI, drug-susceptibility testing, and coordinated multidisciplinary management are essential for achieving favorable outcomes. In some patients, a combination of medical and surgical treatments may result in substantial recovery, even when the disease is severe at the outset.

## Limitations

The primary limitation in managing this patient was the absence of treatment guidelines for individuals with TB-IRIS and tuberculous ventriculitis, particularly the lack of specific criteria for surgical intervention. By leveraging the experiences documented in the limited prior literature, consulting with seasoned colleagues, and meticulously monitoring the patient’s symptoms, we endeavored to perform surgical intervention solely to alleviate quadriparesis resulting from spinal cord compression and to manage ventriculitis symptoms without resorting to additional surgical procedures or ventricular drainage.

## Conclusion

The case details a rare case of drug-resistant CNS-TB with unusual neurological complications, including TB-IRIS, ventriculitis, and spinal tuberculoma. It also highlights knowledge gaps, particularly in managing ventriculitis, which can lead to severe complications if not addressed properly. Physicians should screen all cases of CNS-TB for drug resistance and closely monitor for TB-IRIS, drug-induced hepatitis, and HAI. If these events occur, appropriate interventions should be considered. Future research should focus on systematic reviews and meta-analyses addressing drug resistance, drug-induced hepatitis, and treatment strategies for tuberculous ventriculitis.

## Data Availability

All data underlying the results are available as part of the manuscript, and no additional source data are required.

## Research registration unique identifying number (UIN)

This case report is part of a broader study investigating central nervous system infections, which was registered and approved under the ethical code: IR.AJAUMS.REC.1402.85 (Date: 17 July 2023).
